# Evaluating LLMs in non-metastatic melanoma care: a comparative analysis

**DOI:** 10.3389/fmed.2026.1908569

**Published:** 2026-09-07

**Authors:** Xizhi Wu, Wangyan Zhong, Wanli Ye, Xueying Jin, Jianqing Zhang, Lili Wu

**Affiliations:** 1 Department of Radiation Oncology, Shaoxing People’s Hospital, The First Hospital of Shaoxing University, Shaoxing, Zhejiang, China; 2 Department of Oncology, Shaoxing People’s Hospital, The First Hospital of Shaoxing University, Shaoxing, Zhejiang, China; 3 Department of Dermatology, Shaoxing People’s Hospital, The First Hospital of Shaoxing University, Shaoxing, Zhejiang, China

**Keywords:** clinical decision support, cutaneous melanoma, large language models, TNM staging, treatment standardization

## Abstract

**Background:**

Malignant melanoma is an aggressive skin cancer with rising incidence. Accurate early staging and standardized treatment are crucial for prognosis. This study evaluates seven large language models (LLMs)-including GPT-5.2, Gemini-3.1, and medically enhanced models-in assisting non-metastatic melanoma management amid clinical complexities.

**Methods:**

Employing a prospective, simulated expert-blinded design, 59 virtual cases across TNM stages, ages, and comorbidities were assessed. Multiple senior oncologists independently evaluated model outputs using a 6-point Likert scale for staging accuracy, treatment rationality, and protocol standardization.

**Results:**

GPT-5.2 (5.56 ± 1.12) and Gemini-3.1 (5.25 ± 1.3) achieved the highest staging accuracy, while AntAngelMed performed worst (2.93 ± 1.67). Performance declined significantly in complex Stage III cases. GPT-5.2 and Gemini-3.1 also led in treatment rationality, showing stability, whereas model performances converged in early stages but diverged in advanced ones. Gemini-3.1 excelled in protocol standardization (5.17 ± 0.57), though some models posed risks like insufficient surgical margin recommendations.

**Conclusion:**

Leading LLMs demonstrate potential for high-quality melanoma management but exhibit inconsistent performance influenced by architecture and case complexity, with reduced reliability in advanced stages. Future tools require risk-stratified guidelines and real-world validation to improve patient outcomes.

## Introduction

Malignant melanoma, as a highly aggressive cutaneous malignancy, has shown a concerning, sustained increase in global incidence over the past few decades, posing a severe public health challenge (1). Failure to achieve early detection and standardized intervention in this disease readily leads to lymph node metastasis and distant organ dissemination, significantly worsening patient prognosis, threatening lives, and imposing a heavy economic burden on families and healthcare systems (2). In the clinical management of non-metastatic malignant melanoma, precise tumor staging is the cornerstone for developing individualized treatment strategies, currently based primarily on the internationally recognized TNM staging system and the latest guidelines from authoritative bodies such as the National Comprehensive Cancer Network (NCCN) and the Chinese Society of Clinical Oncology (CSCO). However, despite clear guideline directives, the actual clinical decision-making process faces numerous complex obstacles: as the disease stage advances, especially into intermediate and locally advanced stages, the spectrum of treatment options expands exponentially, involving multiple dimensions such as defining surgical margins, determining indications for sentinel lymph node biopsy (SLNB), and weighing the pros and cons of adjuvant therapies (3–5). Furthermore, the rapid evolution of medical knowledge makes it difficult for clinicians to keep pace with all updated recommendations in real-time. Coupled with objective differences in resource allocation, treatment philosophies, and accumulated experience among different medical institutions, significant heterogeneity exists in the management strategies for patients at the same stage across different centers (6). This lack of standardization in care may directly impact patient survival outcomes (7). Therefore, there is an urgent need to explore novel technological tools that can assist clinical decision-making and enhance the consistency and standardization of diagnosis and treatment to address increasingly complex clinical demands.

In recent years, the rapid development of artificial intelligence technology, particularly the rise of large language models (LLMs), has provided new perspectives and potential solutions for improving the aforementioned clinical challenges. Previous studies have preliminarily confirmed that general-purpose LLMs demonstrate remarkable capabilities in tasks such as medical knowledge question-answering, medical record summarization, and basic diagnostic reasoning (8–10). However, existing literature has predominantly focused on evaluations in general medical scenarios or single tasks. There remains a scarcity of systematic research comparing the performance of various latest-generation general-purpose LLMs and specialized medical-enhanced LLMs across the complete diagnostic and therapeutic workflow for a specific cancer type like malignant melanoma. Particularly critical is the current lack of in-depth exploration based on stratified analysis using detailed TNM staging, which prevents a clear understanding of whether model performance dynamically changes with increasing disease complexity. Although theoretically, LLMs possess the potential to rapidly integrate the latest guidelines and cutting-edge literature by leveraging their vast training data, thereby providing standardized references for complex clinical scenarios, their true efficacy in highly specialized areas such as tumor staging judgment, multi-dimensional treatment decision-making, and the standardization of specific plan execution still requires validation through rigorous empirical research. Filling this knowledge gap holds significant scientific importance for revealing the feasibility boundaries and limitations of advanced AI models in melanoma diagnosis and treatment, thereby promoting their translation into clinical application. A recent comparative study on melanoma did evaluate several LLMs against established patient information resources in answering patient questions, highlighting their potential in providing personalized and complete responses, though also noting concerns regarding accuracy and readability that need addressing (11).

Given this, our study designed a prospective, simulated clinical case-based evaluation study, aiming to establish a standardized evaluation framework that closely aligns with real-world clinical practice. We meticulously constructed 59 simulated cases covering diverse disease stages (from early Stage I to locally advanced Stage III), varied demographic characteristics, and complex comorbidities to ensure the comprehensiveness and representativeness of the testing scenarios. On this basis, this study selected seven representative LLMs as the core evaluation subjects, including five mainstream general-purpose LLMs and two specialized models enhanced with specific medical data, to comprehensively cover the main types in the current technological ecosystem. The core advantage of this methodology lies in its strict controlled variable design: standardized prompt engineering and novel conversational task settings eliminate input bias, ensuring fairness and comparability in the evaluation across different models (12); simultaneously, the introduction of multiple senior oncology experts for independent blind review and the use of pre-designed multi-dimensional Likert scales to quantify the model outputs maximize the professionalism and objectivity of the evaluation results (13, 14). This multi-level, multi-dimensional evaluation strategy can not only provide a horizontal comparison of overall performance but also deeply analyze the nuanced performance differences of the models across different clinical subgroups.

The specific objectives of this study extend beyond mere performance ranking; we are committed to a deeper analysis of the comprehensive performance characteristics and underlying patterns of LLMs in assisting with the diagnosis and treatment of non-metastatic malignant melanoma. We will focus on evaluating model performance across three critical dimensions: accuracy of tumor staging, reasonableness of treatment decisions, and standardization of plan execution, with particular attention to the moderating effect of disease stage on model performance. Through meticulous stratified analysis, this study seeks to answer a core scientific question: as disease complexity increases, does the reasoning capability of LLMs remain stable or exhibit significant fluctuations? Do models with different architectures and training strategies possess specific areas of strength or systematic weaknesses when confronted with high-difficulty clinical decisions? The answers to these questions will provide valuable empirical data for understanding the application potential of artificial intelligence in oncology. Furthermore, this study aims to define the reliability thresholds of various models in assisting clinical decision-making under current technological conditions, thereby laying a solid methodological foundation and theoretical basis for the future development of safer and more reliable clinical decision support systems.

Given this, in the face of the standardization challenges and decision-making complexity inherent in the diagnosis and treatment of non-metastatic malignant melanoma, a systematic evaluation of the practical efficacy of LLMs appears particularly urgent. Through innovative simulated case design and a rigorous expert blind review mechanism, this study conducts, for the first time, a comprehensive and in-depth assessment of multiple cutting-edge LLMs within the same framework. We expect that this research will not only clarify the true capabilities of current mainstream models in melanoma staging and treatment decision-making, but also reveal the key factors influencing their performance, particularly the dynamic impact of disease stage on model output. The findings will provide a scientific basis for clinicians to select appropriate AI-assisted tools, while also guiding AI developers in optimizing model algorithms and enhancing their robustness in complex oncology scenarios. Ultimately, the discoveries from this study have the potential to promote the deep integration of artificial intelligence technology with clinical oncology, contributing to the realization of more precise, standardized, and accessible melanoma diagnosis and treatment services, thereby improving clinical outcomes for a broad patient population.

## Materials and methods

### Research design and case construction

The methodological core of this study was to establish a clinically grounded benchmarking framework for evaluating large language models (LLMs) in dermatologic oncology. Rather than using verbatim electronic health records, we constructed 59 de-identified clinical vignettes​ adapted from real-world non-metastatic melanoma cases managed at our institution.

Each vignette employed a structured adaptation protocol: medically essential decision-making parameters—including Breslow thickness, ulceration status, primary tumor location, regional lymph node findings, and comorbidity severity—were abstracted from source records, while all potentially identifying narrative details were fictionalized. For instance, demographic descriptors (e.g., “38-year-old software engineer” or “52-year-old teacher”) and subjective timelines were deliberately altered to create novel, non-identifiable scenarios that simulate authentic outpatient encounters.

To ensure systematic coverage, the 59 cases were distributed across a test matrix of AJCC Stages I–III, varied age strata, and representative comorbidity profiles (Supplementary File). Using a standardized prompt (Supplementary File), each LLM was tasked with providing a complete clinical response, including: (1) clinical TNM staging, (2) systemic/local treatment regimen selection, and (3) specific therapeutic details (e.g., surgical margins, sentinel lymph node biopsy indications). This design allows rigorous assessment of LLM reasoning fidelity against real clinical decision nodes without compromising patient privacy.

### Chatbot selection and data collection

We investigated the performance of seven large language models, including five general-purpose LLMs (ChatGPT-5.2, Gemini-3.1, Deepseek-V3.2, Qwen3.5-Plus, and GLM-5) and two medically enhanced LLMs (Baichuan-M3-Plus, AntAngelMed). All models were accessed via their official web interfaces using default reasoning modes and standard temperature settings, with web-search functionalities explicitly disabled to prevent external knowledge contamination. The complete standardized prompt template is provided in Supplementary File. For each clinical case, queries were manually entered from March 15 to April 1, 2026, and responses were collected directly from the interface. To mitigate memory effects on the results, a new session was created for each scenario, ensuring that all LLMs and cases used the same prompt.

### Blinded review and evaluation

All responses were anonymized and evaluated by a blinded expert panel from April 2 to April 15, 2026. The experts were asked to assess the answers in three aspects: accuracy of diagnostic staging, rationality of treatment decisions, and standardization of specific implementation (Supplementary Table 1).

Each clinical case was independently reviewed by five experts with at least ten years of experience in oncology treatment. Using a pre-designed 6-point Likert scale, the experts independently scored the staging accuracy, rationality of treatment decisions, and standardization of the implementation plan provided by the large language models. Scoring was primarily based on the Chinese Society of Clinical Oncology (CSCO) and National Comprehensive Cancer Network (NCCN) guidelines, while also referencing the latest clinical research findings according to predefined criteria.

In addition to directly comparing the overall performance of the five general-purpose large language models and the two medically enhanced models in the diagnosis and treatment of non-metastatic malignant melanoma, this study further conducted a detailed analysis of the 59 clinical cases stratified by TNM stage. The cases covered TNM stages IA, IB, IIA, IIB, IIC, IIIA, IIIB, IIIC, and IIID. Detailed distributions of staging are provided in Supplementary Table 2.

### Statistical analysis

Statistical analyses and visualizations were performed using Python (SciPy and Pingouin libraries). For each metric—Diagnostic Accuracy, Therapeutic Appropriateness, and Standardization of Implementation (all scored on a 1–6 Likert scale)—descriptive statistics including mean and standard deviation were calculated for each large language model.

Given the ordinal nature of the Likert-scale ratings and the comparison of more than two independent models, inter-model differences were assessed using the Kruskal–Wallis H test. When the overall test was statistically significant (*p* < 0.05), Dunn's *post-hoc* test with Bonferroni correction​ was applied to evaluate pairwise differences between individual models.

Inter-rater agreement among the multiple clinical evaluators was quantified using Fleiss’ kappa coefficient. For all tests, statistical significance was set at *p* < 0.05.

To complement *p*-values and assess practical relevance, 95% confidence intervals (CIs) and Cohen's d (standardized effect size) were calculated for key inter-model comparisons, with AntAngelMed set as the baseline. Effect sizes were interpreted using predefined clinical significance thresholds (Small: d < 0.5; Medium: 0.5 ≤ d < 0.8; Large: 0.8 ≤ d < 1.3; Very Large: d ≥ 1.3).

### Ethical considerations

Given that this study utilized hypothetical clinical scenarios derived from actual melanoma cases and did not include any real patient data or demographic characteristics, it applied for and was granted an ethics review exemption in accordance with institutional guidelines governing human subjects research.

## Results

### Accuracy of tumor staging

**Figure 1:**
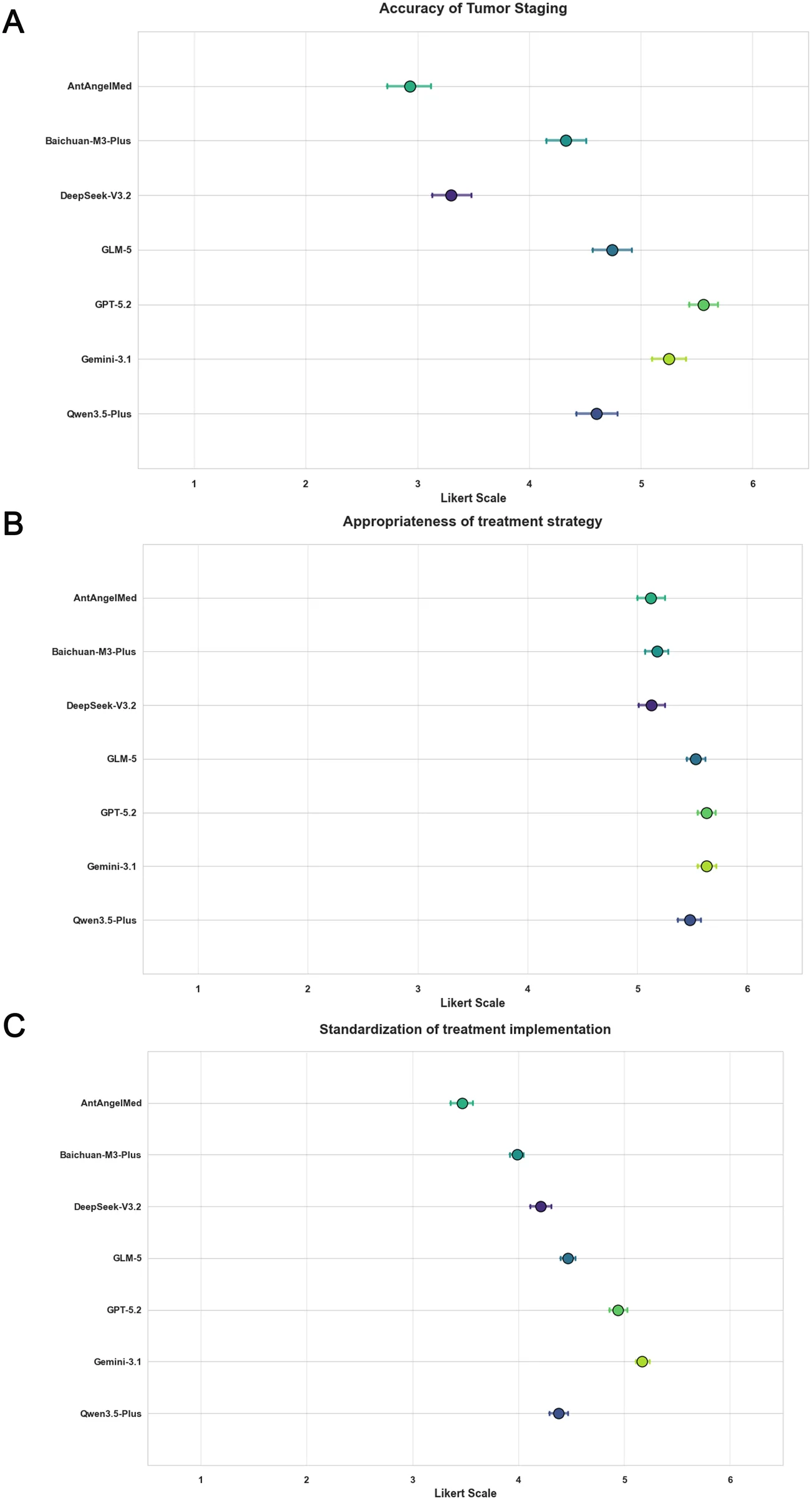
Comparative performance of large language models (LLMs) across three clinical tasks: **(A)** tumor staging accuracy, **(B)** treatment strategy appropriateness, and **(C)** treatment implementation standardization. Data are presented as mean scores with 95% confidence intervals (error bars) on a 1–6 Likert scale. Points represent group means; models are ordered horizontally to facilitate visual comparison of inter-model variability.

Regarding the clinical staging of malignant melanoma, expert evaluations demonstrated nearly perfect agreement (Kappa = 0.9, *P* < 0.001). Overall, significant performance differences were observed among the different models. GPT-5.2 achieved the highest mean accuracy score (5.56 ± 1.12), indicating it had the best overall accuracy in this evaluation, with a relatively concentrated score distribution (small standard deviation). Gemini-3.1 performed next best, with a mean accuracy of 5.25 ± 1.30. Three models (GLM-5, Qwen3.5-Plus, and Baichuan-M3-Plus) formed a middle tier, with mean accuracy scores of 4.74 ± 1.51, 4.60 ± 1.61, and 4.33 ± 1.56, respectively. DeepSeek-V3.2 had a lower mean accuracy of 3.30 ± 1.50. The AntAngelMed model recorded the lowest mean accuracy in this evaluation (2.93 ± 1.67) and also exhibited the largest standard deviation, suggesting that not only does its accuracy require improvement for this task, but the consistency (or stability) of its responses is relatively weak (Table 1, Figure 1A). Pairwise comparisons revealed that the differences in staging accuracy among all the large language models were statistically significant.

**Table 1:** Performance of large language models (LLMs) in malignant melanoma staging on a Likert scale, reported as mean, standard deviation (SD), and 95% confidence interval (CI). Effect sizes (Cohen's d) are provided relative to the baseline model (AntAngelMed).

Model	Mean	SD	CI_lower	CI_upper	Cohen's d
DeepSeek-V3.2	3.3	1.5	3.13	3.48	0.23 (Small)
Qwen3.5-Plus	4.6	1.61	4.42	4.79	1.02 (Large)
GLM-5	4.74	1.51	4.57	4.92	1.14 (Large)
Baichuan-M3-Plus	4.33	1.56	4.15	4.51	0.87 (Medium)
AntAngelMed	2.93	1.67	2.73	3.12	-
GPT-5.2	5.56	1.12	5.43	5.69	1.85 (Very Large)
Gemini-3.1	5.25	1.3	5.1	5.4	1.55 (Very Large)

A detailed assessment and analysis were conducted to evaluate the accuracy performance of each large language model across specific TNM stages (ⅠA to ⅢD) of malignant melanoma. The results show that models GPT-5.2 and Gemini-3.1 consistently demonstrated superior or competitive accuracy in most stages. GPT-5.2 achieved a perfect score of 6.0 in stages ⅠB, ⅡA, ⅡB, ⅡC, ⅢC, and ⅢD, indicating robust and top-tier performance. Gemini-3.1 also obtained perfect scores in stages ⅠB, ⅡB, and ⅡC, but its score dropped to 3.77 in stage ⅢD. In stage ⅢB, the overall scores of all models were notably lower. Although GPT-5.2 (4.3 points) and Gemini-3.1 (4.62 points) led the group, no model scored above 5 points, suggesting that task complexity or data characteristics may vary by stage. The performance of models Qwen3.5-Plus, GLM-5, and Baichuan-M3-Plus showed fluctuations, while DeepSeek-V3.2 exhibited even greater variability—scoring higher in early stages (e.g., 5.0 in stage ⅠA) but lower in several advanced stages. The AntAngelMed model consistently received the lowest accuracy scores across all stages, with particularly low scores in stage ⅢB (1.85), stage ⅢC (1.8), and stage ⅢD (1.6), indicating the most unstable performance (Table 2). This suggests that the model faces significant challenges in handling the nuances of more advanced melanoma stages or in meeting specific evaluation criteria.

**Table 2:** Accuracy scores of AI models across TNM stages.

Stage	DeepSeek-V3.2	Qwen3.5-Plus	GLM-5	Baichuan-M3-Plus	AntAngelMed	GPT-5.2	Gemini-3.1
ⅠA	5	4.5	4.6	4.5	4.5	4.5	5
ⅠB	2.6	4.8	5.4	3	3.08	6	6
ⅡA	3.5	4	5	4.5	3.6	6	5.5
ⅡB	3.17	4.5	5	4.43	4.73	6	6
ⅡC	3	6	4.8	5.4	4.2	6	6
ⅢA	4.46	4.91	5.57	4.77	2.17	5.57	5.14
ⅢB	3.12	3.75	3.92	4.18	1.85	4.3	4.62
ⅢC	2.48	5	4.76	3.34	1.8	6	5.28
ⅢD	2.6	4.43	3.57	5.77	1.6	6	3.77

### Appropriateness of treatment strategy

Regarding treatment decisions for non-metastatic malignant melanoma, expert evaluations demonstrated high agreement (*κ* = 0.71, *p* < 0.001). Descriptive statistics indicated that the mean scores of all models fell within a high and concentrated range. GPT-5.2 and Gemini-3.1 achieved the highest mean scores (both 5.63) with the smallest standard deviations (0.70 and 0.73, respectively), indicating that they provided the most appropriate treatment strategies with the most stable performance. No statistically significant difference was found between these two top-performing models (*P* = 0.241). Furthermore, the differences between GPT-5.2/Gemini-3.1 and models such as Qwen3.5-Plus and GLM-5 were also not significant. However, significant performance gaps existed between GPT-5.2/Gemini-3.1 and other models, including DeepSeek-V3.2, Baichuan-M3-Plus, and AntAngelMed (Table 3, Figure 1B).

**Table 3:** Performance of large language models (LLMs) in appropriateness of treatment strategy on a Likert scale, reported as mean, standard deviation (SD), and 59% confidence interval (CI). Effect sizes (Cohen's d) are provided relative to the baseline model (AntAngelMed).

Model	Mean	SD	CI_lower	CI_upper	Cohen's d
DeepSeek-V3.2	5.13	1.07	5.01	5.25	0.01 (Small)
Qwen3.5-Plus	5.48	0.9	5.37	5.58	0.36 (Small)
GLM-5	5.53	0.74	5.45	5.62	0.44 (Small)
Baichuan-M3-Plus	5.18	0.93	5.07	5.28	0.06 (Small)
AntAngelMed	5.12	1.08	5	5.25	-
GPT-5.2	5.63	0.7	5.55	5.71	0.56 (Medium)
Gemini-3.1	5.63	0.73	5.55	5.72	0.55 (Medium)

Further, we conducted a more granular assessment and analysis of the performance of each large language model regarding treatment strategies across specific clinical TNM stages (ⅠA to ⅢD) of non-metastatic malignant melanoma. The study found that GPT-5.2 and Gemini-3.1 exhibited excellent and consistent performance across all stages, with their scores consistently ranking at the top, demonstrating reliable decision stability. In contrast, the performance of DeepSeek-V3.2 and AntAngelMed showed greater fluctuations. For instance, DeepSeek-V3.2 performed best in stage ⅠA (6.00) but saw its score plummet to the lowest in stage ⅡA (3.93); AntAngelMed achieved high scores in stages ⅠA and ⅠB (both 6.00) yet dropped sharply to 3.93 in stage ⅡB. This indicates that the adaptability and response consistency of such models to specific clinical scenarios need improvement. Furthermore, differences in disease complexity across stages significantly influenced the performance distribution of the models. In the early stages (ⅠA-ⅠB), all models performed excellently (scores mostly ≥5), with several achieving perfect scores, indicating a high degree of consensus and appropriateness in the treatment strategies provided by the models for early-stage disease. Moving into the intermediate stages (ⅡA-ⅡC), model performance began to diverge significantly. For example, in stage ⅡA, scores ranged from 3.93 (DeepSeek-V3.2) to 6.00 (GPT-5.2), revealing a noticeable gap and suggesting that increased disease complexity started to challenge the models’ discrimination and adaptation capabilities. In the advanced stages (ⅢA-ⅢD), no single model could consistently maintain absolute dominance, and the competitive landscape became differentiated. Specifically, in stage ⅢA, Gemini-3.1 scored the highest (5.97); in stage ⅢB, GLM-5 and Baichuan-M3-Plus stood out (5.90, 5.88); in stage ⅢC, Gemini-3.1 and GPT-5.2 led (5.64, 5.52); and in stage ⅢD, GPT-5.2 achieved the highest score (5.67). This reveals that when handling complex late-stage cases, different models may have variations in their knowledge focus and reasoning strategies (Table 4).

**Table 4:** Appropriateness scores of treatment strategies by AI models across TNM stages.

Stage	DeepSeek-V3.2	Qwen3.5-Plus	GLM-5	Baichuan-M3-Plus	AntAngelMed	GPT-5.2	Gemini-3.1
ⅠA	6	6	5.67	5.67	6	5.67	5.33
ⅠB	4.88	6	6	6	6	6	6
ⅡA	3.93	4.33	5.13	4.53	5.77	6	5.77
ⅡB	5.7	5.87	5.7	4.7	3.93	5.47	5.7
ⅡC	4.76	5.52	5.2	4.48	4.64	5.6	5.92
ⅢA	5.6	5.91	5.83	5.6	4.94	5.83	5.97
ⅢB	5.65	5.15	5.9	5.88	5.25	5.22	5.18
ⅢC	4.62	5.42	5.1	4.82	4.92	5.52	5.64
ⅢD	5	5.33	5.43	4.8	4.8	5.67	5.43

### Standardization of treatment implementation

Regarding the standardization of treatment implementation for non-metastatic malignant melanoma, expert evaluations demonstrated high agreement (*κ* = 0.65, *p* < 0.001). Gemini-3.1 achieved the highest standardization score (5.17 ± 0.57), indicating it performed best in adhering to or simulating standardized treatment protocols, with high output stability (smallest standard deviation). GPT-5.2 ranked second (4.94 ± 0.72), showing a minor gap from the leading model. GLM-5, Qwen3.5-Plus, and DeepSeek-V3.2 formed a middle-performance tier, with scores of 4.47 ± 0.62, 4.38 ± 0.75, and 4.21 ± 0.83, respectively. It is noteworthy that GLM-5 exhibited a relatively low standard deviation within this tier, suggesting better output consistency. Baichuan-M3-Plus scored close to but below the middle tier (3.99 ± 0.55); its low standard deviation indicates that while its performance was not outstanding, it was relatively stable. AntAngelMed received the lowest standardization score (3.47 ± 0.94) and had the largest standard deviation, indicating that for this task, not only does its accuracy require improvement, but the variability or uncertainty in its outputs is also the highest (Table 5, Figure 1C). *Post-hoc* pairwise comparisons indicated statistically significant inter-model variability in the capability to standardize treatment implementation across all evaluated LLMs (*p* < 0.05 for all comparisons).

**Table 5:** Performance of large language models (LLMs) in standardization of treatment implementation on a Likert scale, reported as mean, standard deviation (SD), and 59% confidence interval (CI). Effect sizes (Cohen's d) are provided relative to the baseline model (AntAngelMed).

Model	Mean	SD	CI_lower	CI_upper	Cohen's d
DeepSeek-V3.2	4.21	0.83	4.11	4.31	0.83 (Large)
Qwen3.5-Plus	4.38	0.75	4.29	4.47	1.07 (Large)
GLM-5	4.47	0.62	4.4	4.54	1.26 (Large)
Baichuan-M3-Plus	3.99	0.55	3.92	4.05	0.68 (Medium)
AntAngelMed	3.47	0.94	3.36	3.57	-
GPT-5.2	4.94	0.72	4.86	5.03	1.76 (Very Large)
Gemini-3.1	5.17	0.57	5.11	5.24	2.19 (Very Large)

Further, we conducted a more granular assessment and analysis of the standardization of specific implementation performance for each large language model across individual clinical stages (ⅠA to ⅢD) of non-metastatic malignant melanoma. The study found that although Gemini-3.1 led overall, its performance was surpassed by GPT-5.2 in certain specific, complex stages. For instance, in stage ⅡC, Gemini-3.1 scored 5.28, while GPT-5.2 scored 5.52; in stage ⅢC, the former scored 4.9, and the latter 5.12. This suggests that even top-performing models may have relative weaknesses in their ability to standardize treatment implementation in specific complex clinical scenarios. In contrast, the medically enhanced model AntAngelMed consistently received lower scores across stages, indicating potential systematic shortcomings in adhering to standardized treatment implementation, with a tendency towards non-standard recommendations such as “insufficient margins.” Furthermore, the performance of models Qwen3.5-Plus and DeepSeek-V3.2 showed the greatest fluctuations across different stages, reflecting inadequacies in their output stability (Table 6).

**Table 6:** Standardization scores of treatment implementation by AI models across malignant melanoma stages.

Stage	DeepSeek-V3.2	Qwen3.5-Plus	GLM-5	Baichuan-M3-Plus	AntAngelMed	GPT-5.2	Gemini-3.1
ⅠA	5	5.43	5	4.23	3.57	5.07	5.6
ⅠB	3.64	3.96	4.6	3.92	3.48	4.6	4.96
ⅡA	3.83	4.57	4.5	4.13	3.33	4.83	5.4
ⅡB	4.47	4.5	4.23	4.1	4.07	5.47	5.5
ⅡC	4.4	4.48	4.24	4	3.48	5.52	5.28
ⅢA	3.94	4.11	4.26	4.26	3.97	4.71	5.11
ⅢB	3.45	4.35	4.53	4.03	3.3	4.47	5
ⅢC	4.5	4.1	4.36	3.68	3.2	5.12	4.9
ⅢD	4.83	4.1	4.5	3.4	3.17	4.73	5

## Discussion

The global incidence of malignant melanoma, a highly aggressive skin cancer, continues to rise (1). Without early, precise intervention, its progression increases metastatic risk and healthcare burdens (2). While clinical management relies on the TNM staging system and guidelines for personalized care, advancing stages bring complex treatment options and rapid guideline updates, creating significant decision-making challenges and practice variations that hinder standardization (5). In the non-metastatic stage, accurate staging and treatment are vital for prognosis. However, unequal medical resource distribution and delayed physician knowledge updates impede clinical consistency, highlighting an urgent need for efficient, standardized decision-support tools to optimize the clinical workflow.

To the best of our knowledge, this study is among the first to utilize a highly realistic evaluation framework to systematically probe the capabilities of LLMs in non-metastatic melanoma management. Beyond merely comparing the horizontal discrepancies between general and medical-enhanced models, our approach innovatively introduced a longitudinal, stage-stratified analysis. This design allowed us to uncover a nuanced picture: while leading models achieved remarkable accuracy and stability in early-stage cases, their performance exhibited notable volatility in complex, advanced scenarios, with varied models dominating at different specific stages. These findings suggest that although state-of-the-art LLMs show initial promise for delivering high-quality therapeutic advice, their real-world clinical deployment demands a cautious approach. Specifically, clinicians must be acutely aware of the heterogeneity risks tied to disease staging. Ultimately, rather than expecting a single “one-size-fits-all” model, our data lay a vital empirical foundation for designing next-generation, tiered, and highly robust clinical decision support systems tailored to specific disease phases.

The high inter-rater agreement among experts (Fleiss’ Kappa = 0.9) established a robust baseline by confirming the objective biological criteria of melanoma staging compared to more subjective soft tissue tumors (15, 16). Utilizing this reliable benchmark, evaluation results demonstrated that in terms of staging accuracy, general-purpose models GPT-5.2 (5.56 ± 1.12) and Gemini-3.1 (5.25 ± 1.3) achieved optimal performance, whereas the specialized medical-enhanced model AntAngelMed (2.93 ± 1.67) performed the worst. We unexpectedly found that general-purpose LLMs (e.g., GPT-5.2) outperformed specialized medical models, likely because the latter “overfit” to narrow guideline texts at the expense of reasoning on raw anatomical phenotypes in borderline cases (17). Furthermore, it is noteworthy that the majority of models exhibited a significant performance decline when processing late-stage (Stage III) complex cases**.** This deterioration, particularly evident in Stage ⅢB, is driven by non-linear biological complexities—such as occult nodal involvement—which disrupt purely text-based reasoning chains, paralleling the known limitations of PET-CT in detecting micro-metastases without multi-modal pathological evidence (18, 19). Ultimately, these divergent patterns underscore how general semantic comprehension and specialized knowledge retrieval play distinct roles in complex clinical reasoning.

In evaluating treatment rationality, GPT-5.2 and Gemini-3.1 similarly maintained a leading and stable position. While models showed convergent performance in early cases, their advantageous domains diverged in advanced cases. This significant divergence in Stage II profoundly mirrors the ongoing clinical controversies surrounding high-risk Stage II melanoma (e.g., SLNB indication and adjuvant immunotherapy), which stem from the difficulties of integrating molecular biomarkers into precise risk stratification (20, 21). Top-performing models successfully internalized modern, multi-factorial risk algorithms (incorporating mitotic rate, ulceration, and gene expression), whereas poorer performers relied on outdated protocols, failing to deliver personalized, evidence-based recommendations. Conversely, in advanced stages, the varied strengths among models suggest that no single LLM can optimize treatment across all subtypes; this aligns with findings that multi-agent systems simulating multidisciplinary teams (MDTs) outperform single-perspective models, highlighting the necessity for multi-dimensional clinical reasoning (22). Furthermore, the hallucinations or logical gaps observed in late-stage recommendations likely stem from insufficient training data coverage of rare subtypes or emerging therapies like bispecific antibodies (23). Ultimately, Stage II divergence serves as a critical litmus test for an LLM's capacity to capture dynamic risk stratification updates, while late-stage inconsistencies underscore the imperative for multi-modal knowledge integration in tackling complex oncological challenges.

Evaluation of treatment execution standardization revealed a critical gap: overall, Gemini-3.1 (5.17 ± 0.57) held a slight edge in this dimension, but several models still harbored potential clinical risks. While models proposed sound macro-strategies, they frequently failed to translate these into precise micro-operational parameters (e.g., surgical margins, lymph node dissection extents). For instance, some models exhibited potential clinical risks such as recommending insufficient surgical margins, with AntAngelMed exhibited a systematic tendency toward this flaw. This mechanism-level flaw likely stems from pretraining on unstructured clinical notes containing historical, non-standard practices, leading to “averaged” outputs rather than strict adherence to NCCN/CSCO thickness-based margin guidelines (24). Conversely, Gemini-3.1's superior standardization may reflect robust instruction-following capabilities and enhanced attention mechanisms for structured guidelines, allowing it to enforce precise numerical constraints similar to specialized deep learning models extracting exact radiomic features (25). Nevertheless, even leading models showed standardization fluctuations in complex Stage IIC/IIIC contexts, where multi-morbidity disrupts their reasoning chains and causes deviations from standard protocols. These discrepancies carry significant clinical weight, as unconstrained generative AI risks propagating substandard practices—particularly concerning given that non-compliant surgical margins are directly correlated with increased local recurrence rates (26). Ultimately, reasonable decision-making does not automatically guarantee execution standardization, highlighting the stringent constraint-satisfaction requirements necessary for reliable clinical AI.

Several limitations of this study must be acknowledged. First, our evaluation relied on meticulously constructed virtual cases; while designed to closely mimic clinical reality, they lack the multimodal noise and inherent complexity typical of real-world data, potentially overestimating the models’ robustness in actual, uncontrolled clinical environments. Second, the relatively limited sample size of 59 non-metastatic melanoma cases, combined with a strict reliance on current guidelines and expert subjective scoring, fails to adequately capture cutting-edge, individualized treatment strategies or rare subtypes, thereby limiting the generalizability of our findings. Third, while prompt engineering details were partially disclosed, a more critical caveat inherent to generative AI research is the rapid evolution of proprietary model architectures. Our results represent a “snapshot-in-time” evaluation of the specific model versions accessed between March and April 2026; performance may shift substantially with future releases, and readers should interpret findings as reflective of these particular iterations rather than immutable model capabilities.

Fourth, our current comparator panel did not include Anthropic's Claude models; future benchmarking should incorporate additional frontier models (e.g., Claude 3.5) to broaden the scope of inference. Most crucially, this study exclusively focused on the quality of the decision-making process, without correlating model outputs with hard clinical endpoints—such as survival or recurrence rates—which remain the ultimate metrics for validating the clinical translational value of artificial intelligence.

## Conclusion

In conclusion, utilizing a multidimensional expert-blinded evaluation framework, this study systematically delineates the performance stratification and critical determinants of various LLMs in the clinical management of non-metastatic malignant melanoma. Our central finding indicates that while leading general-purpose models (e.g., GPT-5.2, Gemini-3.1) exhibit near-expert reliability in staging, therapeutic decision-making, and execution standardization, their performance substantially deteriorates in advanced complex scenarios, with certain specialized medical models unexpectedly underperforming. These insights offer pivotal empirical grounding for the future development of clinical decision support systems (CDSS). Specifically, LLMs can function as highly efficient, standardized references for early or routine cases, yet their application in high-risk, advanced stages necessitates stringent integration with human expert review. Moving forward, research priorities should include the development of dynamic, multi-turn interactive evaluation paradigms and the investigation of model ensemble strategies to bolster robustness across complex staging dilemmas. Ultimately, the definitive validation of these AI tools hinges on prospective real-world studies demonstrating tangible improvements in patient clinical outcomes.

## Data Availability

The original contributions presented in the study are included in the article/Supplementary Material, further inquiries can be directed to the corresponding author.
