# Supplementary material for: Evaluating LLMs in non-metastatic melanoma care: a comparative analysis
Source: Front Med (Lausanne). 2026 Sep 7;13:1908569. doi: 10.3389/fmed.2026.1908569 (PMC13590381; doi:10.3389/fmed.2026.1908569)
Supplement: Supplementary file 1 [file Table1.docx]

Table S1.Definition of accuracy in diagnostic staging, appropriateness of treatment decisions, standardization of treatment implementation.

| Accuracy in diagnostic staging with feedback  was categorised as | 1 Completely wrong  Deviation by more than two stages, leading to a fundamental error in treatment direction.  2 Mostly wrong  Multiple inaccuracies in T/N/M subgroupings.  3 Partially correct  T/N/M subgroupings are correct, but the derived clinical stage is incorrect.  4 Mostly correct  Staging is essentially accurate, with minor discrepancies in defining the extent of involvement (e.g., specific lymph node groups, degree of adjacent organ invasion).  5 Almost entirely correct  Staging is highly accurate and largely consistent with postoperative pathology or gold-standard diagnostic findings.  6 Correct  Staging (including histology, subtype, molecular classification, and TNM) is entirely precise and aligns perfectly with the gold standard. |
| --- | --- |
| Appropriateness of  treatment strategy | 1 Completely inappropriate  The strategy is entirely contrary to established guidelines.  2 Inappropriate  The strategy deviates from the key principles of guidelines and lacks an evidence-based rationale.  3 Largely inappropriate  The general direction of treatment is partially correct, but with major flaws.  4 Largely appropriate  The overall strategy aligns with guideline standards, but the specific regimen is non-optimal.  5 Appropriate  The treatment strategy is highly rational, strictly adheres to guideline recommendations, and is logically designed and well-targeted.  6 Perfectly appropriate  The treatment strategy is optimal, integrating the latest evidence-based medicine with individual patient characteristics for a tailored, precision approach. |
| Standardization of treatment implementation | 1 Completely non-standard  The selected technique or medication is obsolete, contraindicated, or clearly unsuitable, potentially causing rapid progression or iatrogenic harm.  Surgical Margins:  Excision margins < 0.5 cm for any invasive melanoma without justification due to anatomical constraints.  Routine use of Mohs micrographic surgery for invasive melanoma (outside of specific cases like lentigo maligna).  Staging / Medication:  Recommending "observation only" for Stage IIIA+ patients without adjuvant therapy.  Prescribing BRAF/MEK inhibitors (e.g., Dabrafenib/Trametinib) for a BRAF wild-type patient.  Using outdated chemotherapy (e.g., Dacarbazine) as first-line for metastatic disease when immunotherapy/targeted therapy is indicated.  Critical Omission: Performing wide local excision without evaluating clinically positive lymph nodes via FNA or core biopsy.  2 Non-standard  The main modality is correct, but critical errors in key parameters exist that significantly alter prognosis.  Surgical Margins:  Breslow thickness > 2 mm with a planned 1 cm margin (should be 2 cm).  Melanoma in situassigned a 2 cm margin (overtreatment; standard is 0.5–1 cm).  Sentinel Lymph Node Biopsy (SLNB):  Failure to offer SLNB for high-risk T1b (e.g., 0.8 mm with ulceration) without documented reason.  Routinely performing SLNB for low-risk T1a (<0.8 mm, no ulceration).  Adjuvant Therapy:  Recommending high-dose interferon for Stage IIB/IIC instead of standard-of-care PD-1 inhibitors (Pembrolizumab/Nivolumab).  3 Largely non-standard  The framework is correct, but deviations in parameters or incomplete application weaken the outcome.  Surgical Margins:  Correct margin chosen, but no consideration for anatomical location (e.g., using 2 cm on the face without discussing functional reconstruction or staged excision).  Ambiguous description (e.g., "approx. 1 cm") without ensuring true pathological clearance.  Adjuvant Therapy:  Choosing adjuvant PD-1 therapy but planning a 6-month course (standard is 1 year).  Initiating BRAF/MEK inhibitors without baseline cardiac/ophthalmic exams or clear management rules for pyrexia.  Advanced Disease:  Treating a BRAF-mutant patient with single-agent immunotherapy without acknowledging the higher evidence level of combination targeted therapy (D+T).  4 Largely standard  The primary treatment meets guidelines, but lacks individualized details or supportive care. It is "textbook" but not "patient-specific."  Surgical Margins:  States "margin based on Breslow thickness" but fails to specify the exact measurement for this patient (e.g., "1–2 cm").  Adjuvant Therapy:  Correctly recommends Pembrolizumab but omits toxicity management (e.g., thyroid function monitoring, rash management).  Supportive Care:  Focuses on anti-tumor therapy but ignores melanoma-specific surveillance (e.g., secondary skin cancer screening, lymphedema prevention education).  5 Standard  Precise and compliant descriptions of the primary technique, dosage, and cycles. Supportive therapy details are present but could be slightly more robust.  Surgical Margins:  Clear specification: "Breslow 1.5 mm, no ulceration. Planned 1 cm margin. Considering secondary intention healing for distal digit."  Adjuvant Therapy:  Clear specification: "Stage IIIB, BRAF V600E+. Recommend Dabrafenib 150 mg BID + Trametinib 2 mg daily for 12 months. Baseline ECHO/CXR completed."  Follow-up:  Includes specific timelines (e.g., imaging at 6 and 12 months) and lab tests (LDH, Skin exams).  6 Perfectly standard  Provides precise, traceable, and comprehensive descriptions across all aspects. Ready for immediate execution.  Surgical Margins:  "Melanoma in situ, digit 4. Planned 0.5 cm margin. If subungual matrix involved, plan for disarticulation at DIP joint. Frozen section pending."  MDT Integration:  "Per MDT discussion: Adjuvant Radiotherapy 50 Gy in 25 fractions to neck basin due to extracapsular extension (ENE)." |

Table S2. Distribution of cases by cancer stage.

| Stage | Number of Cases | Percentage |
| --- | --- | --- |
| ⅠA | 6 | 10.2 |
| ⅠB | 5 | 8.5 |
| ⅡA | 6 | 10.2 |
| ⅡB | 6 | 10.2 |
| ⅡC | 5 | 8.4 |
| ⅢA | 7 | 11.9 |
| ⅢB | 8 | 13.5 |
| ⅢC | 10 | 16.9 |
| ⅢD | 6 | 10.2 |
