# Supplementary material for: Evaluating LLMs in non-metastatic melanoma care: a comparative analysis
Source: Front Med (Lausanne). 2026 Sep 7;13:1908569. doi: 10.3389/fmed.2026.1908569 (PMC13590381; doi:10.3389/fmed.2026.1908569)
Supplement: Supplementary file 2 [file Table2.doc]

**Prompt**

I am a physician in the Department of Medical Oncology at a tertiary Grade‑A hospital, specializing in multimodal comprehensive cancer treatment. Please provide expert clinical recommendations for the following case in strict accordance with the latest clinical practice guidelines. The response must use precise medical terminology and explicitly cover: **clinical TNM staging, treatment regimen, and specific therapeutic details**. Be concise, directly address key points, and avoid redundant statements.

**Case**

1. Patient, 38-year-old male, software engineer. ECOG PS 0. Comorbidities: Mild fatty liver. Chief complaint: A “birthmark” on the right shoulder with uneven color and blurred margins for the past 6 months. No ulceration, no pruritus. Clinical examination and regional lymph node ultrasound showed no abnormalities. Biopsy pathology: Malignant melanoma, Breslow thickness 0.3mm, without ulceration.

Patient, 52-year-old female, teacher. ECOG PS 0. Comorbidities: Allergic rhinitis. Chief complaint: A long-standing black mole on the posterior left lower leg gradually enlarging to approximately 4 cm over the past year. Clinical examination and regional lymph node ultrasound showed no abnormalities. Biopsy pathology: Malignant melanoma, Breslow thickness 0.5mm, without ulceration.Patient, 29-year-old female, outdoor tour guide. ECOG PS 0. Comorbidities: None. Chief complaint: A new irregular brown patch on the right cheek for 6 months, with recent darkening. PET-CT and sentinel lymph node biopsy (requested by the patient due to occupational anxiety) showed no metastasis. Biopsy pathology: Malignant melanoma, Breslow thickness 0.6mm, without ulceration.Patient, 41-year-old female, accountant. ECOG PS 0. Comorbidities: Chronic gastritis. Chief complaint: A black mole on the left sole gradually enlarging. Clinical examination and regional lymph node ultrasound showed no abnormalities. Biopsy pathology: Malignant melanoma, Breslow thickness 0.4mm, but ulceration formation observed microscopically.Patient, 33-year-old male, fitness coach. ECOG PS 0. Comorbidities: None. Chief complaint: A mole on the back occasionally bleeding after friction during workouts for 3 months. Clinical examination and regional lymph node ultrasound showed no abnormalities. Biopsy pathology: Malignant melanoma, Breslow thickness 0.9mm, without ulceration.

Patient, 67-year-old male, retired worker. ECOG PS 1. Comorbidities: Hypertension. Chief complaint: A black nodule appearing in the hairless area on the top of the head for 8 months, with occasional mild tenderness. Biopsy pathology: Malignant melanoma, Breslow thickness 0.7mm, without ulceration. Head MRI and cervical lymph node ultrasound showed no metastasis.

Patient, 60-year-old male, fisherman. ECOG PS 0. Comorbidities: Chronic solar dermatitis. Chief complaint: A black patch on the left auricle for 2 years, slowly enlarging. Chest and abdominal CT, head MRI, and cervical lymph node ultrasound showed no metastasis. Biopsy pathology: Malignant melanoma, thickness 1.2mm, without ulceration. Sentinel lymph node negative.

Patient, 57-year-old female, accountant. ECOG PS 0. Comorbidities: Hyperlipidemia. Chief complaint: A subcutaneous nodule on the right neck for 6 months, with darkening of the overlying skin. Chest and abdominal CT, head MRI, and cervical lymph node ultrasound showed no metastasis. Biopsy pathology: Malignant melanoma, thickness 1.8mm, without ulceration, sentinel lymph node negative.Patient, 49-year-old female, nurse. ECOG PS 0. Comorbidities: Type 2 diabetes. Chief complaint: A mole on the right neck gradually enlarging, with darkening of the overlying skin. Chest and abdominal CT, head MRI, and cervical lymph node ultrasound showed no metastasis. Biopsy pathology: Malignant melanoma, thickness 1.5mm, without ulceration, SLNB negative.Patient, 70-year-old male, calligrapher. ECOG PS 1. Comorbidities: Cataracts, mild osteoporosis. Chief complaint: A pigmented patch on the right dorsal hand gradually enlarging and darkening. Chest and abdominal CT, head MRI, and superficial lymph node ultrasound showed no metastasis. Biopsy pathology: Malignant melanoma, thickness 1.5mm, without ulceration, SLNB negative. Patient, 80-year-old male, retired cadre. ECOG PS 2. Chief complaint: A black patch on the posterior right thigh gradually enlarging. Chest and abdominal CT, head MRI, and superficial lymph node ultrasound showed no metastasis. Biopsy pathology: Malignant melanoma, thickness 1.9mm, without ulceration, SLNB negative. Patient, 55-year-old male, taxi driver. ECOG PS 1. Comorbidities: Fatty liver. A pigmented patch on the right dorsal hand for 1 year, with recent surface ulceration and crusting. Chest and abdominal CT, MRI, and superficial lymph node ultrasound showed no metastasis. Biopsy pathology: Malignant melanoma, thickness 1.1mm, with ulceration, SLNB negative. Patient, 55-year-old male, driver. ECOG PS 1. Comorbidities: Hypothyroidism. Chief complaint: A mole on the left waist rapidly enlarging with pruritus for 2 months. Chest and abdominal CT, MRI, and superficial lymph node ultrasound showed no metastasis. Biopsy pathology: Malignant melanoma, thickness 1.5mm, with ulceration, SLNB negative.

Patient, 63-year-old female, retired teacher. ECOG PS 2. Comorbidities: Osteoporosis. Chief complaint: A giant black patch on the posterior right thigh with recurrent ulceration for 1 year. Chest and abdominal CT, head MRI, and superficial lymph node ultrasound showed no metastasis. Biopsy pathology: Malignant melanoma, thickness 2.0mm, with ulceration, SLNB negative.

Patient, 51-year-old male, gardener. ECOG PS 1. Comorbidities: None. A black mole on the left forearm for many years, with obvious elevation in the past 6 months. Chest and abdominal CT, head MRI, and superficial lymph node ultrasound showed no metastasis. Biopsy pathology: Malignant melanoma, thickness 2.3mm, without ulceration, SLNB negative.

Patient, 70-year-old female, lawyer. ECOG PS 0. Comorbidities: Hypertension (well-controlled with medication). An “age spot” on the right zygomatic region for 20 years, significantly darkening and elevating over the past 3 months, with occasional pruritus. Cervical and systemic superficial lymph node ultrasound showed no significant enlargement; contrast-enhanced chest/abdomen CT and head MRI showed no distant metastasis. Biopsy pathology: (Right zygomatic region) Cutaneous malignant melanoma. Microscopically, tumor cells were spindle and epithelioid, depth of invasion (Breslow thickness) 2.6mm, no surface ulceration, with scant lymphocytic infiltration, SLNB negative.

Patient, 75-year-old male, retired construction worker. ECOG PS 1. Comorbidities: None. A black mole on the left dorsal hand for 40 years, a “fleshy bump” growing over the past 6 months, bleeding easily upon touch. Left axillary and epitrochlear lymph node ultrasound showed no abnormalities; whole-body PET-CT and cranial MRI showed no extra-cutaneous or visceral metastasis. Biopsy pathology: (Left dorsal hand) Acral malignant melanoma. Depth of invasion (Breslow thickness) 2.1mm, without ulceration, SLNB negative.

Patient, 58-year-old female, retired worker. ECOG PS 1. Comorbidities: Hypertension. Chief complaint: A mass on the left scapular region with pain and exudation for 6 months. Whole-body PET-CT and cranial MRI showed no extra-cutaneous or visceral metastasis; superficial lymph node ultrasound showed no abnormalities. Biopsy pathology: Malignant melanoma, thickness 2.5mm, with ulceration, SLNB negative.

Patient, 47-year-old male, salesperson. Comorbidities: Fatty liver. An irregular pigmented patch on the abdomen, growing slowly for 2 years. Superficial lymph node ultrasound showed no abnormalities; whole-body PET-CT and cranial MRI showed no extra-cutaneous or visceral metastasis. Biopsy pathology: Malignant melanoma, thickness 3.0mm, with ulceration, SLNB negative.

Patient, 71-year-old male, retired cadre. Comorbidities: Coronary heart disease. A cauliflower-like mass on the scalp for 1 year, bleeding easily. Superficial lymph node ultrasound showed no abnormalities; whole-body PET-CT and cranial MRI showed no extra-cutaneous or visceral metastasis. Biopsy pathology: Malignant melanoma, thickness 3.8mm, with ulceration, SLNB negative.

Patient, 36-year-old female, nurse. Comorbidities: None. Chief complaint: A rapidly enlarging painless nodule on the right buttock for 1 year. Superficial lymph node ultrasound showed no abnormalities; cranial MRI and chest/abdominal CT showed no extra-cutaneous or visceral metastasis. Biopsy pathology: Thickness 5.5mm, without ulceration, SLNB negative.

Patient, 52-year-old female, accountant. ECOG PS 0. Comorbidities: None. A painless mass on the left lower back for 8 months, significantly enlarging over the past 3 months, accidentally palpated, without pruritus or ulceration. Bilateral axillary and inguinal superficial lymph node ultrasound showed no abnormalities; cranial MRI and whole-body PET-CT showed no extra-cutaneous or visceral metastasis. Biopsy pathology: (Left lower back) Cutaneous malignant melanoma. Depth of invasion (Breslow thickness) 6.2mm, no surface ulceration, SLNB negative.

Patient, 41-year-old female, bank teller. ECOG PS 1. Comorbidities: None. A black patch on the lateral right sole for 2 years, rapidly elevating and forming an induration over the past year; did not seek medical attention as it was painless, non-pruritic, and did not affect walking; recently visited the doctor due to tight shoes. Bilateral inguinal and popliteal lymph node ultrasound showed no enlargement; contrast-enhanced cranial MRI and contrast-enhanced chest/abdominal CT showed no definitive metastasis. Biopsy pathology: (Right sole) Acral malignant melanoma. Depth of invasion (Breslow thickness) 4.8mm, no surface ulceration, SLNB negative.

Patient, 60-year-old female, housewife. ECOG PS 1. Comorbidities: Osteoporosis. Chief complaint: A giant ulcerated mass on the left heel with foul odor for 8 months. Superficial lymph node ultrasound showed no abnormalities; cranial MRI and whole-body PET-CT showed no extra-cutaneous or visceral metastasis. Biopsy pathology: Malignant melanoma, thickness 4.2mm, with ulceration, SLNB negative.

Patient, 65-year-old male, farmer. ECOG PS 1. Comorbidities: Chronic bronchitis. A giant black tumor on the right lower leg with surface ulceration and infection, lasting for 2 years. Bilateral inguinal and popliteal lymph node ultrasound showed no enlargement; contrast-enhanced cranial MRI and contrast-enhanced chest/abdominal CT showed no definitive metastasis. Biopsy pathology: Malignant melanoma, thickness 8.0mm, with ulceration, SLNB negative. Patient, 68-year-old male, coastal fisherman. ECOG PS 1. Comorbidities: Hypertension for 10 years (well-controlled with medication). A black patch on the left heel ulcerating and discharging pus with foul odor for 1.5 years, growing into a fist-sized mass over the past 6 months, unable to wear shoes or walk. Bilateral inguinal and popliteal lymph node ultrasound showed no abnormalities; contrast-enhanced cranial MRI and contrast-enhanced chest/abdominal CT showed no metastasis. Biopsy pathology: (Left heel) Acral malignant melanoma. Depth of invasion (Breslow thickness) 7.5mm, surface ulceration formation, accompanied by massive necrosis and acute/chronic inflammatory cell infiltration, SLNB negative.

Patient, 72-year-old male, retired lumberjack. ECOG PS 1. Comorbidities: Type 2 diabetes for 8 years (oral hypoglycemics, fasting blood glucose fluctuating 7.5-9.0 mmol/L), chronic bronchitis. A “black mole” on the right upper back scratched and failing to heal for a long time over 2 years, rapidly elevating and suppurating over the past 4 months. Bilateral axillary and supraclavicular lymph node ultrasound showed no enlargement; cranial MRI and whole-body PET-CT ruled out extra-cutaneous and visceral metastasis. Biopsy pathology: (Right upper back) Cutaneous malignant melanoma. Depth of invasion (Breslow thickness) 9.2mm, definite ulceration, SLNB negative.

Patient, 63-year-old male, construction mason. ECOG PS 1. Comorbidities: Chronic obstructive pulmonary disease (COPD, GOLD Grade 1), no long-term medication history. Chief complaint: A black mass on the extensor side of the right forearm progressively enlarging with ulceration and bleeding for 2 years. Right epitrochlear and axillary lymph node ultrasound showed no abnormalities; cranial MRI, chest CT, and whole-abdomen contrast-enhanced CT showed no definitive metastasis. Biopsy pathology: (Right forearm) Malignant melanoma. Depth of invasion (Breslow thickness) 8.8mm, ulceration observed, SLNB negative.

Patient, 45-year-old female, clerk. ECOG PS 0. Comorbidities: Chronic gastritis. A black mole on the lateral left lower leg for many years, slightly darkening over the past 6 months, without ulceration or pruritus. Whole-body PET-CT and cranial MRI showed no definitive metastasis. Biopsy pathology: Malignant melanoma, thickness 0.6mm, without ulceration. Sentinel lymph node pathology: 1 lymph node detected in the left inguinal region, showing melanoma micrometastasis with a diameter of about 0.5mm. Genetic testing: BRAF V600 mutation.

Patient, 38-year-old male, software engineer. ECOG PS 0. Comorbidities: Mild fatty liver. Chief complaint: A light black patch appearing on the right shoulder for 1 year. Superficial lymph node ultrasound showed no abnormalities; contrast-enhanced chest/abdomen CT and cranial MRI showed no metastasis. Biopsy pathology: Malignant melanoma, thickness 0.5mm, without ulceration. Sentinel lymph node pathology: 1 lymph node detected in the right axilla, extremely small micrometastasis foci found microscopically. Genetic testing: BRAF V600 mutation. Patient, 52-year-old female, teacher. ECOG PS 0. Comorbidities: Allergic rhinitis. Chief complaint: A black mole on the mid-left lower leg slightly elevating for 6 months. Whole-body imaging (including contrast-enhanced CT of neck, chest, abdomen, pelvis and brain MRI) showed no extra-cutaneous metastasis. Biopsy pathology: Malignant melanoma, thickness 0.5mm, without ulceration. Sentinel lymph node pathology: 2 sentinel lymph nodes detected in the left popliteal fossa, both showing microscopic micrometastasis. Genetic testing: Negative.

Patient, 29-year-old female, outdoor tour guide. ECOG PS 0. Comorbidities: None. Chief complaint: A black mole on the right cheek repeatedly crusting and bleeding for 3 months. Contrast-enhanced head/neck MRI and high-resolution cervical ultrasound showed no definitive enlarged lymph nodes; imaging of other body parts was negative. Biopsy pathology: Primary lesion: (Right cheek) Malignant melanoma, thickness 0.6mm, with ulceration. Sentinel lymph node pathology: 3 sentinel lymph nodes detected in the right parotid region and cervical Level II, all showing micrometastasis foci. Genetic testing: Negative.

Patient, 33-year-old male, fitness coach. ECOG PS 0. Comorbidities: None. Dark spots found on the back for 8 months, no significant changes. Whole-body imaging showed no signs of metastasis. Biopsy pathology: (Back) Malignant melanoma, thickness 0.9mm, without ulceration. Sentinel lymph node pathology: 1 lymph node detected in the left axilla, showing micrometastasis. Genetic testing: Negative.

Patient, 60-year-old male, fisherman. ECOG PS 1. Comorbidities: Chronic solar dermatitis. A black-brown patch on the left auricle for 1 year, with mild surface scaling over the past 3 months. Contrast-enhanced head/neck CT and whole-body PET-CT showed no lymphadenopathy or distant metastasis. Biopsy pathology: (Left auricle) Malignant melanoma, thickness 1.2mm, without ulceration. Sentinel lymph node pathology: 1 lymph node detected in the left postauricular region, showing micrometastasis. Genetic testing: Negative.

Patient, 29-year-old female, outdoor tour guide. ECOG PS 0. Comorbidities: None. Chief complaint: A black mole on the right cheek repeatedly crusting and bleeding for 3 months. Contrast-enhanced head/neck MRI and high-resolution cervical ultrasound showed no definitive enlarged lymph nodes; imaging of other body parts was negative. Biopsy pathology: Primary lesion: (Right cheek) Malignant melanoma, thickness 1.2mm, without ulceration. Sentinel lymph node pathology: 3 sentinel lymph nodes detected in the right parotid region and cervical Level II, all showing micrometastasis foci. Genetic testing: Negative.

Patient, 45-year-old female, clerk. ECOG PS 0. Comorbidities: Chronic gastritis. Chief complaint: A black patch on the left lower leg gradually enlarging for 2 months. Superficial lymph node ultrasound showed no abnormalities; chest/abdomen CT and brain MRI showed no distant metastasis. Biopsy pathology: Primary lesion pathology is malignant melanoma, thickness 0.6mm, without ulceration. SLNB indicated 1 dominant lymph node metastasis. Genetic testing: Negative.

Patient, 52-year-old female, teacher. ECOG PS 0. Comorbidities: Allergic rhinitis. A black patch on the left lower leg darkening for 3 months; within a 2cm range of the lesion, 2 black papules about 0.3cm in diameter were visible. Bilateral inguinal and popliteal lymph node ultrasound was negative; whole-body imaging showed no metastasis. Biopsy pathology: Primary lesion pathology malignant melanoma, thickness 0.5mm, without ulceration. Papule pathology confirmed melanoma microsatellite metastasis. Genetic testing: Negative.

Patient, 41-year-old female, accountant. ECOG PS 0. Comorbidities: Chronic gastritis. Chief complaint: Left sole ulceration and crusting with pain for 2 months. Auxiliary examination: Whole-body PET-CT and brain MRI showed no definitive metastasis. Biopsy pathology: (Left sole) Malignant melanoma, thickness only 0.4mm, but definite surface ulceration observed; SLNB revealed 1 inguinal lymph node metastasis. Genetic testing: Negative.

Patient, 41-year-old female, accountant. ECOG PS 0. Comorbidities: Chronic gastritis. Chief complaint: Left sole ulceration and crusting with pain for 2 months. Auxiliary examination: Whole-body PET-CT and brain MRI showed no definitive metastasis. Biopsy pathology: (Left sole) Malignant melanoma, thickness only 0.4mm, but definite surface ulceration observed; SLNB revealed 1 inguinal lymph node metastasis. Genetic testing: Negative.

Patient, 40-year-old male, chef. ECOG PS 0. Comorbidities: None. A black mole on the right forearm enlarging with ulceration for 8 months; a palpable enlarged lymph node about 2.0cm in the right axilla. Auxiliary examination: Chest/abdomen CT and brain MRI showed no metastasis. Biopsy pathology: Axillary lymph node puncture confirmed metastasis. Primary lesion biopsy pathology: Melanoma, thickness 2.3mm, without ulceration. Genetic testing: Negative.

Patient, 38-year-old male, software engineer. ECOG PS 0. Comorbidities: Mild fatty liver. A melanin patch on the right shoulder enlarging for 4 months, 2 tiny black spots seen at 1.5cm; whole-body imaging showed no distant metastasis. Primary lesion biopsy pathology showed thickness 0.3mm, without ulceration; shoulder black spots excised, pathology confirmed as satellite lesions. Concurrent SLNB revealed 1 axillary lymph node micrometastasis. Genetic testing: Negative.

Patient, 58-year-old male, park greening administrator. ECOG PS 0. Comorbidities: Hypertension (well-controlled with medication). Chief complaint: A black mole on the left forearm ulcerating and bleeding for 3 months. Left axillary high-resolution ultrasound showed no abnormal lymph nodes; contrast-enhanced cranial MRI and contrast-enhanced chest/abdomen/pelvis CT showed no distant metastasis (M0). Biopsy pathology: Primary lesion: (Left forearm) Malignant melanoma. Microscopically measured depth of invasion 1.6mm, definite surface ulceration. Sentinel lymph node biopsy (SLNB): 3 sentinel lymph nodes detected in the left axilla, 2 of which showed microscopic melanoma micrometastasis. Genetic testing: BRAF V600 mutation.

Patient, 45-year-old female, primary school teacher. ECOG PS 0. Comorbidities: Mild iron deficiency anemia (no medication, diet adjusting). A painless black patch on the right lower back for 1 year, slowly elevating. Right axillary and supraclavicular lymph node ultrasound showed no abnormalities; cranial MRI and whole-body PET-CT showed no lymphadenopathy or distant visceral metastasis. Biopsy pathology: Primary lesion: (Right lower back) Malignant melanoma. Microscopically measured depth of invasion 2.8mm, no surface ulceration; Sentinel lymph node biopsy (SLNB): 4 sentinel lymph nodes detected in the right axilla, 3 of which showed microscopic micrometastasis. Genetic testing: BRAF V600 mutation.

Patient, 70-year-old male, farmer. ECOG PS 1. Chronic bronchitis. A painless ulcerated mass on the top of the head for 1.5 years; a hard lymph node about 2.5cm palpable in the right postauricular area, fixed. Auxiliary examination: Chest/abdomen CT and brain MRI showed no distant metastasis. Initial biopsy pathology: Primary lesion malignant melanoma, thickness 6.5mm, with ulceration. Postauricular lymph node puncture confirmed metastasis. Genetic testing: BRAF V600 mutation.

Patient, 55-year-old female, housewife. ECOG PS 0. No comorbidities. A black mole on the left lower leg ulcerating and bleeding for 6 months; 2 fused enlarged lymph nodes palpable in the left inguinal region. Auxiliary examination: Cranial MRI and whole-body PET-CT ruled out metastasis. Biopsy pathology: Primary lesion malignant melanoma, thickness 2.8mm, with ulceration. Inguinal lymph node biopsy positive, 2 palpable clinically. Genetic testing: Negative.

Patient, 45-year-old male, interior designer. ECOG PS 0. Mild fatty liver. A black spot on the right back slowly enlarging for 1 year, no other discomfort. Ultrasound showed normal axillary lymph node morphology; whole-body CT showed no metastasis. Biopsy pathology: Primary lesion malignant melanoma, thickness 2.2mm, without ulceration. SLNB: 5 sentinel lymph nodes detected in the right axilla, 4 were microscopic micrometastasis. Genetic testing: Negative.

Patient, 35-year-old female, university teacher. ECOG PS 0. No comorbidities. A black mole on the left shoulder slightly elevating for 6 months. No lymph nodes palpable in the left axilla, ultrasound negative. Imaging showed no metastasis. Biopsy pathology: Malignant melanoma, primary lesion thickness 0.9mm, without ulceration; SLNB performed, 4 lymph nodes with micrometastasis detected in the left axilla. Genetic testing: Negative.

Patient, 60-year-old male, construction worker. ECOG PS 1. Hypertension. A black mole on the right forearm enlarging for 1 year; several fused enlarged lymph nodes palpable in the right axilla. Auxiliary examination: Contrast-enhanced CT suggested axillary mass invading surrounding fat space. No distant metastasis. Biopsy pathology: Malignant melanoma, primary lesion thickness 1.5mm, without ulceration. Lymph node puncture indicated metastasis. Genetic testing: Negative.

Patient, 50-year-old female, chef. ECOG PS 0. No comorbidities. A painful black mass on the left heel for 8 months; 4 mutually fused lymph nodes palpable in the left inguinal region. Biopsy pathology: Malignant melanoma, primary lesion thickness 4.5mm, without ulceration. Inguinal lymph node biopsy positive, and mutually fused. Genetic testing: Negative.

Patient, 48-year-old male, postman. ECOG PS 0. No comorbidities. A black mole on the left lower leg ulcerating for 6 months; 3 satellite lesions visible within 2cm around the primary lesion; 2 enlarged lymph nodes palpable in the left inguinal region. Initial biopsy pathology: Malignant melanoma, primary lesion thickness 1.8mm, with ulceration. Inguinal lymph node puncture positive. Skin nodules confirmed as melanoma satellite metastasis. Genetic testing: BRAF V600 mutation.

Patient, 58-year-old male, construction worker. ECOG PS 1. Hyperlipidemia. A black mole on the right lower leg ulcerating and bleeding for 6 months; 3 scattered black subcutaneous small nodules (satellite lesions) visible on the skin within a 3cm range around the primary lesion. A hard enlarged lymph node about 2.5cm palpable in the right inguinal region, fixed and painless. Auxiliary examination: Cranial MRI and whole-body PET-CT confirmed lesions only in the local right lower leg and right inguinal region, no visceral or distant metastasis (M0). Biopsy pathology: Primary lesion: Malignant melanoma, invasion thickness 1.8mm, no surface ulceration. Inguinal lymph node puncture: Confirmed melanoma metastasis (clinically dominant metastasis). Skin nodule biopsy: Confirmed melanoma satellite metastasis. Genetic testing: BRAF V600 mutation.

Patient, 65-year-old female, farmer. ECOG PS 0. Chronic gastritis. A black patch with recurrent ulceration and crusting on the left heel for 1 year; no enlarged lymph nodes palpable/detected on clinical examination and ultrasound in bilateral inguinal regions. Auxiliary examination: Contrast-enhanced CT of chest, abdomen, pelvis and brain MRI showed no distant metastasis (M0). Biopsy pathology: Primary lesion: Malignant melanoma, invasion thickness 5.2mm, definite ulceration observed microscopically. Sentinel lymph node biopsy (SLNB): 2 sentinel lymph nodes detected in the left inguinal region, both showing tiny melanoma metastatic foci microscopically. Genetic testing: Negative.

Patient, 48-year-old female, middle school teacher. ECOG PS 0. No comorbidities. A black mole on the lateral right thigh slightly elevating for 6 months, scratched on the surface in the past month, not healing well. Auxiliary examination: Whole-body imaging showed no definitive metastatic foci. Biopsy pathology: Primary lesion: Malignant melanoma, invasion thickness 2.6mm, definite ulceration formation observed microscopically. Sentinel lymph node biopsy (SLNB): 1 sentinel lymph node detected in the right inguinal region, extremely tiny metastatic foci visible microscopically. Genetic testing: BRAF V600 mutation.

Patient, 65-year-old male, farmer. ECOG PS 1. Chronic bronchitis. A giant ulcerated mass on the right lower leg for 2 years; 4 hard lymph nodes palpable in the right inguinal region (diameter 1.0-1.5cm, fairly mobile, not fused). Auxiliary examination: Lower extremity ultrasound suggested 4 enlarged lymph nodes in the inguinal region (slightly irregular morphology, but not breaking through the capsule); cranial MRI and whole-body PET-CT ruled out lung, liver, brain and other visceral metastasis; lactate dehydrogenase (LDH) 210 U/L. Primary lesion pathology: Malignant melanoma, invasion depth 8.2mm, definite ulceration observed microscopically; Right inguinal lymph node dissection: 5 lymph nodes retrieved, 4 of which were microscopic micrometastasis. Genetic testing: Negative.

Patient, 58-year-old female, retired teacher. ECOG PS 0. Osteoporosis. A 5cm ulcerated black patch on the left heel for 1 year; 4 hard lymph nodes palpable in the left popliteal fossa (diameter 1.0-1.2cm, good mobility). Auxiliary examination: Lower extremity ultrasound suggested 4 enlarged lymph nodes in the popliteal fossa (slightly abnormal morphology, no fusion); cranial MRI and whole-body PET-CT showed no metastasis; LDH 195 U/L (normal). Pathology and genetics: Primary lesion: Malignant melanoma, invasion depth 6.8mm, with ulceration; Left popliteal SLNB: 4 lymph nodes all showing microscopic micrometastasis; Genetic testing: Negative.

Patient, 62-year-old male, driver. ECOG PS 1 (due to mass pressure sensation). Hypertension. A 4cm ulcerated black patch on the right thigh for 1 year; a mass of about 4cm×3cm palpable in the left inguinal region (hard texture, fixed and adherent to surrounding skin and adipose tissue, loss of mobility). Auxiliary examination: Contrast-enhanced inguinal MRI showed several lymph nodes fusing into a cluster, breaking through the capsule and invading surrounding fat space; whole-body CT showed no visceral metastasis; LDH 205 U/L (normal). Pathology and genetics: Primary lesion: Malignant melanoma, invasion depth 6.5mm, with ulceration; Right inguinal lymph nodes: Fused into a cluster, microscopically tumor cells breaking through the lymph node capsule and invading surrounding adipose tissue; Genetic testing: BRAF V600E mutation.

Patient, 55-year-old female, self-employed. ECOG PS 0. No comorbidities. A 6cm ulcerated mass on the left buttock for 1 year; a fused cluster mass palpable in the left inguinal region (fixed, adherent to surrounding fascia). Auxiliary examination: Pelvic MRI suggested several inguinal lymph nodes fusing, invading surrounding muscle tissue; cranial MRI and whole-body PET-CT showed no metastasis; LDH 220 U/L (normal). Pathology and genetics: Primary lesion: Malignant melanoma, invasion depth 7.2mm, with ulceration; Left inguinal lymph nodes considered metastasis; Genetic testing: BRAF V600E mutation.

58. Patient, 70-year-old male, fisherman. ECOG PS 1 (due to ulcer consumption). Chronic bronchitis. A 10cm giant ulcer on the right buttock for 1.5 years; 3 satellite lesions visible within a 3cm range around the primary lesion (diameter 0.5-1.0cm, black subcutaneous nodules); 2 enlarged lymph nodes palpable in the right inguinal region (diameter 2.0-2.5cm, hard and fixed). Auxiliary examination: Pelvic CT showed inguinal lymphadenopathy, satellite lesions confined to surrounding the primary lesion; cranial MRI and whole-body PET-CT showed no metastasis; LDH 225 U/L (normal). Pathology and genetics: Primary lesion: Malignant melanoma, invasion depth 9.1mm, with ulceration; Right inguinal lymph nodes: Needle biopsy confirmed metastasis; Buttock satellite lesions: Biopsy confirmed melanoma metastasis; Genetic testing: KIT exon13 mutation.

59. Patient, 60-year-old female, teacher. ECOG PS 0. No comorbidities. A 7cm ulcerated black patch on the left lower leg for 1 year; 2 enlarged lymph nodes palpable in the left popliteal fossa (diameter 2.0-3.0cm, hard and fixed); 2 satellite lesions visible within a 2cm range around the primary lesion on the left lower leg (diameter 0.6-0.8cm, flat black spots). Auxiliary examination: Lower extremity CT suggested popliteal lymphadenopathy, satellite lesions confined to the lower leg; cranial MRI and whole-body PET-CT showed no metastasis; LDH 215 U/L (normal). Pathology and genetics: Primary lesion: Malignant melanoma, invasion depth 8.3mm, with ulceration; Left popliteal lymph nodes: Biopsy confirmed metastasis;
